# Using Application Programming Interfaces to Access Google Data for Health Research: Protocol for a Methodological Framework

**DOI:** 10.2196/16543

**Published:** 2020-07-06

**Authors:** Anne Zepecki, Sylvia Guendelman, John DeNero, Ndola Prata

**Affiliations:** 1 The Wallace Center for Maternal, Child, and Adolescent Health School of Public Health University of California, Berkeley Berkeley, CA United States; 2 Department of Electrical Engineering and Computer Sciences College of Engineering University of California, Berkeley Berkeley, CA United States

**Keywords:** Google, search data, infodemiology, infoveillance, infodemic, reproductive health, abortion, birth control, Google Trends, APIs

## Abstract

**Background:**

Individuals are increasingly turning to search engines like Google to obtain health information and access resources. Analysis of Google search queries offers a novel approach, which is part of the methodological toolkit for infodemiology or infoveillance researchers, to understanding population health concerns and needs in real time or near-real time. While searches predominantly have been examined with the Google Trends website tool, newer application programming interfaces (APIs) are now available to academics to draw a richer landscape of searches. These APIs allow users to write code in languages like Python to retrieve sample data directly from Google servers.

**Objective:**

The purpose of this paper is to describe a novel protocol to determine the top queries, volume of queries, and the top sites reached by a population searching on the web for a specific health term. The protocol retrieves Google search data obtained from three Google APIs: Google Trends, Google Health Trends (also referred to as Flu Trends), and Google Custom Search.

**Methods:**

Our protocol consisted of four steps: (1) developing a master list of top search queries for an initial search term using Google Trends, (2) gathering information on relative search volume using Google Health Trends, (3) determining the most popular sites using Google Custom Search, and (4) calculating estimated total search volume. We tested the protocol following key procedures at each step and verified its usefulness by examining search traffic on *birth control* in 2017 in the United States. Two separate programmers working independently achieved similar results with insignificant variation due to sample variability.

**Results:**

We successfully tested the methodology on the initial search term *birth control*. We identified top search queries for *birth control*, of which *birth control pill* was the most popular and obtained the relative and estimated total search volume for the top queries: relative search volume was 0.54 for the pill, corresponding to an estimated 9.3-10.7 million searches. We used the estimates of the proportion of search activity for the top queries to arrive at a generated list of the most popular websites: for the pill, the Planned Parenthood website was the top site.

**Conclusions:**

The proposed methodological framework demonstrates how to retrieve Google query data from multiple Google APIs and provides thorough documentation required to systematically identify search queries and websites, as well as estimate relative and total search volume of queries in real time or near-real time in specific locations and time periods. Although the protocol needs further testing, it allows researchers to replicate the steps and shows promise in advancing our understanding of population-level health concerns.

**International Registered Report Identifier (IRRID):**

RR1-10.2196/16543

## Introduction

Individuals in the United States seeking health information online turn to search engines first. According to a 2012 Pew Internet & American Life survey, 83% of users identified Google as their main search engine [1]. Health questions and concerns are frequently of a sensitive nature, so queries people type privately into a search engine can provide insight about their true health concerns, especially those that they may not be comfortable sharing with their clinician or a research survey. Stephens-Davidowitz has found that these types of searches often capture what people actually “do, think, or want” because people reveal “some very personal things” in constructing their Google queries [2].

The most popular tool for analyzing and aggregating patterns of search data is Google Trends, a public website that has provided real-time and archived data on Google queries by users since 2004 [3]. It has been used to study online behavior on diverse health topics, such as early detection of influenza epidemics [4-6], pertussis outbreak monitoring [7], asthma monitoring [8], and cancer detection [9,10]. The tool has also been used to study public interest in cancer [11,12], suicide assessment [13,14], depression-related information seeking [15], lifestyle-disease surveillance [16], bariatric surgery [17], herpes zoster vaccinations [18], searches for walk-in clinics and emergency departments [19], obesity-related behavior [20], and reproductive health [21-26]. Research using this tool has increased over 20-fold between 2009 and 2018 [27].

From a methodological standpoint, Google Trends has been used to measure web-based interest and variations of this interest over time [7,18,21,28], assess correlations between search queries with other data sources to inform public health and policy [9,26], and to forecast disease occurrence and outbreaks [4,6,29-31]. These applications fall within the emerging field of infodemiology. As first described by Eysenbach, infodemiology is “the science of distribution and determinants of information in an electronic medium, specifically the internet, or in a population, with the ultimate aim to inform public health and public policy” [32]. A related term, infoveillance, has been used where infodemiology methods are employed for surveillance.

While infodemiology was first used to analyze the quality of information on websites (ie, supply side), the scope has expanded to include what people need and their health-seeking behavior (ie, demand side). According to Eysenbach, analyses of information supply and demand both require new methods to measure the epidemiology of information and to examine the relationships between information supply and/or demand and population health [33].

Although Google Trends is an easily accessible tool for analyzing large population search queries, there is no consensus on how to retrieve, organize, and code queries. Researchers have applied inconsistent methodologies when using this tool and interpreting search data, which sometimes has led to questionable or invalid findings and problems with replicability and comparability across studies [34,35]. In response to these shortcomings, Mavragani and Ochoa [35] recently proposed a concise step-by-step methodological framework that describes how to select the appropriate keyword, region, time period, and category for analysis of search queries to ensure the validity of health assessments with the web-based Google Trends tool. This framework, if used appropriately by researchers, should prove useful to ascertain more uniformity and comparability across studies and further our insight into human behavior.

Less noted is that Google data is also available through Google application programming interfaces (APIs). Multimedia Appendix 1 compares the Google Trends website and the API and illustrates their similarities and differences through an example. The Google Trends API can be used as a first step to identify top queries or search terms, and the API can be used in combination with two other Google APIs—Google Health Trends API and the Custom Search API—to extend the researcher’s understanding of search behaviors. The reason to combine APIs is that although the Google Trends website gives insight into search query volume, the additional APIs are needed to relate search intent to individual websites. All three APIs allow users to write code in a programming language such as Python to retrieve sample data directly from Google servers. However, access to the Google Trends API and Health Trends API is restricted to researchers and requires an application to Google.

This article aims at documenting and illustrating a novel protocol for the use of three Google APIs to determine search query volume and individual websites reached by a given population searching using a health-related search term. This protocol is not the only one enabled by these APIs but is appropriate for the stated aim. We draw on examples from our study, which seeks to examine insights obtained from aggregated search queries related to the prevention of pregnancy. Analyses of queries related to birth control are relevant for policy and programmatic efforts because public funding and access to birth control are increasingly under attack in the United States [36]. As access becomes more restrictive, use of the web may become more important in decision making about family planning.

Since there are no accepted methodological standards for the use of Google APIs in academic research, our paper contributes to the systematization of an approach to combining APIs. The proposed methodology allows for a fuller picture of the volume and content of searches we are exploring through the examination of top topics and queries, relative search volume (RSV), top websites visited when searching for these top queries, and estimated volume of searches. The use of multiple APIs also provides multiple methods to estimate key values, ensuring the data obtained are accurate and reliable.

## Methods

### Overview

We obtain key pieces of Google search data by using three Google APIs. Google Trends provides the top search topics and top search queries given an initial search term for a specified time period and location. Google Health Trends generates the RSV for a list of top queries in a specific region and time period. Finally, Google Custom Search provides the list of top websites that people who search using a given initial search term are shown when using the Google search engine. Custom Search gives results at the time of accessing the API, and these results can be specified at the national level.

The Google Health Trends API, previously known as Google Flu Trends, gives normalized RSV across a set of search queries, allowing for more in-depth analysis of the relationships between queries. This RSV refers to the proportion of searches for a specific query as compared to the sum total of searches for a set of queries, and thus differs from the relative search index given by Google Trends, which gives search interest relative to all searches during the specified period of time. Although this proprietary tool is not available via the Google Trends website, it offers benefits to the researcher by providing a clearly defined metric to understand and interpret RSV.

We show that the RSV provided by Google Health Trends can be combined with another trusted data source to estimate total search volume. RSV can also inform estimates of proportions of searches to a given site. To gather information on the top websites displayed on the Google search engine for a specific search term or topic of interest, we can access data through the Custom Search API. Because Custom Search gives results at the time of API access, researchers should plan accordingly and prepare to take regular samples of top sites during the time period of interest. This list of top sites is for the entire country. Evidence shows that the selection, sorting, and ranking criteria of search engines influence online health-information seeking [37]. Custom Search data allow for analysis of content and quality of information that people get online. Thus, while the Google Trends website can determine what information people search for, it cannot determine what information they find. Hence, working with the three APIs enables a more comprehensive analysis than could be completed by using only the Google Trends website.

We developed a simulation protocol that consisted of four steps: identifying a list of search queries for the topics of interest, obtaining RSV, determining top sites for top search queries, and calculating estimated total search volume. We describe these fully in Tables 1-5. We tested the protocol to examine the top queries for *birth control* in the United States in 2017 and created visualizations for each step. We used Python, version 2.7.13 (Python Software Foundation), for all of the API calls. Examples of the Python commands used are shown in Figures MA2-1 to MA2-7 in Multimedia Appendix 2. Multimedia Appendix 3 contains the documentation of the Python package Graphviz [38] and the APIs used.

### Step 1: Developing a List of Search Queries

**Figure 1:**
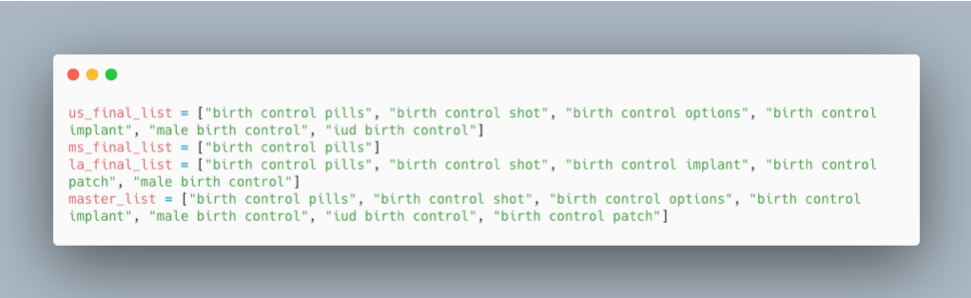
Creation of a master list as visualized in Python for *birth control* in the United States, Mississippi, and Louisiana in 2017.

**Figure 2:**
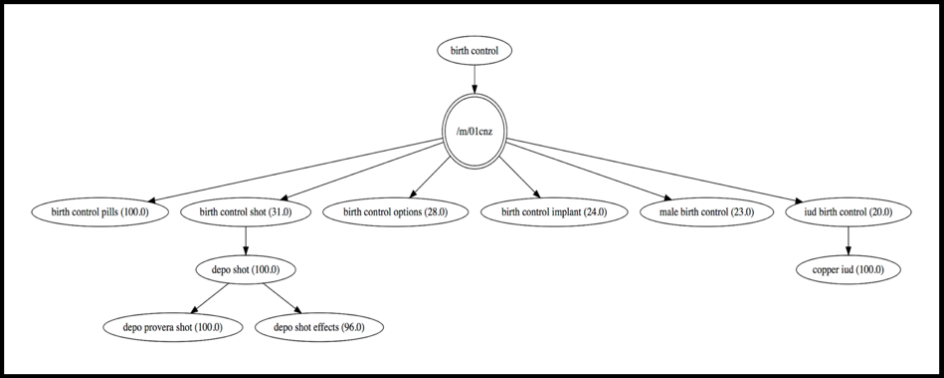
Top queries for *birth control* in the United States in 2017. Single circles in the graph represent search queries, whereas a double circle indicates an overarching topic coded for internal organizational purposes within the Google application programming interface (API) and is not included in the list of top queries. Numbers in parentheses indicate how relation of query to the provided initial search term. iud: intrauterine device.

In the first step, we used the *getTopQueries* function to get the queries most associated with the initial topic of interest during a researcher-specified time period in a researcher-specified geographic region. The *getTopQueries* function can also gather the queries most associated with the previously obtained top queries, referred to as follow-up queries. Top queries are displayed in a graph that illustrates the relationship between queries. More details of the step-by-step procedures carried out are shown in Table 1. Figures 1 and 2 show intermediary Steps 1.3 and 1.5 of the protocol.

**Table 1:** Developing a list of top search queries.

Step	Description
1.1	Begin with a list of regions to explore and a single, broad, initial search term, such as *birth control*.
1.2	For each region, make a request to Google Trends’ *getTopTopics* function to obtain the most searched-for topics for a specific initial search term. The function will return a list of topics that term is most closely related to as well as a value from 0 to 100 that denotes how strongly linked the topic is to the initial term: 100 is the most closely associated and 0 is the least. This list of top topics serves only to validate the top queries by examining similarities between the top topics and top queries.
1.3	Next, make a call to Google Trends’ *getTopQueries* function to get a list of the search queries most related to the initial search term in Step 1.1 for a given region, such as the United States. Each response from the *getTopQueries* method contains a *title*, or query, and a *value* attribute, which is a number from 0 to 100 and represents how related the query is to the provided initial search term in the United States: 100 is the most associated and 0 is the least. The data are presented in the form of a JSON (JavaScript Object Notation)-encoded mapping (see Figure MA2-1 in Multimedia Appendix 2), which can easily be converted into a graph via Python or exported to a CSV (Comma-Separated Values) file. If there are other regions of interest (eg, US states), this step must be repeated for all other regions. Each region will have a *final list* variable that stores all the top queries for that region. Once all final lists are generated for all regions of interest, they will be combined to create a *master list* that includes the top queries for every region of interest (Figure 1 shows an example). Figure MA2-2 in Multimedia Appendix 2 shows a snippet of the Python code.
1.4	For every query generated in Step 1.3, send a request to *getTopQueries* to obtain *follow-up terms*. Only queries with a *value* attribute greater than or equal to 70, as this indicates a high level of correlation between the terms, is added to our *follow-up queries* list. Irrelevant searches relating to pop culture should be manually filtered from results. Step 1.4 should be recursively executed—the *follow-up queries* become the base set at each iteration—until no new queries can be added to the base set. During this step, how each query is related to each other (ie, how a query ended up in our set of queries) should be recorded. This step is terminated when requests to *getTopQueries* do not return unique queries that have not already been received in the simulation for this region.
1.5	Then, generate a graph using the Graphviz package for Python 2.7 [38] that illustrates how the search queries in the *final list* and the *follow-up queries* list are related to one another. As shown in Figure 2, every node in the graph is a search query, and those in the first level will be included in the final list of search queries for the simulation. If a node is encapsulated by a double circle, then this represents an overarching topic coded for internal organizational purposes within the Google application programming interface (API) and is not included in the *final list* or *follow-up queries*. Every direct edge (arrow) in the graph represents a relationship between two search queries (nodes) in the graph. Note that with the current cutoff value of 70 in Step 1.4, there may be other intermediate terms in the graph not captured. Figure MA2-3 in Multimedia Appendix 2 shows the Python function used.

### Step 2: Gathering Information on Search Volume

**Figure 3:**
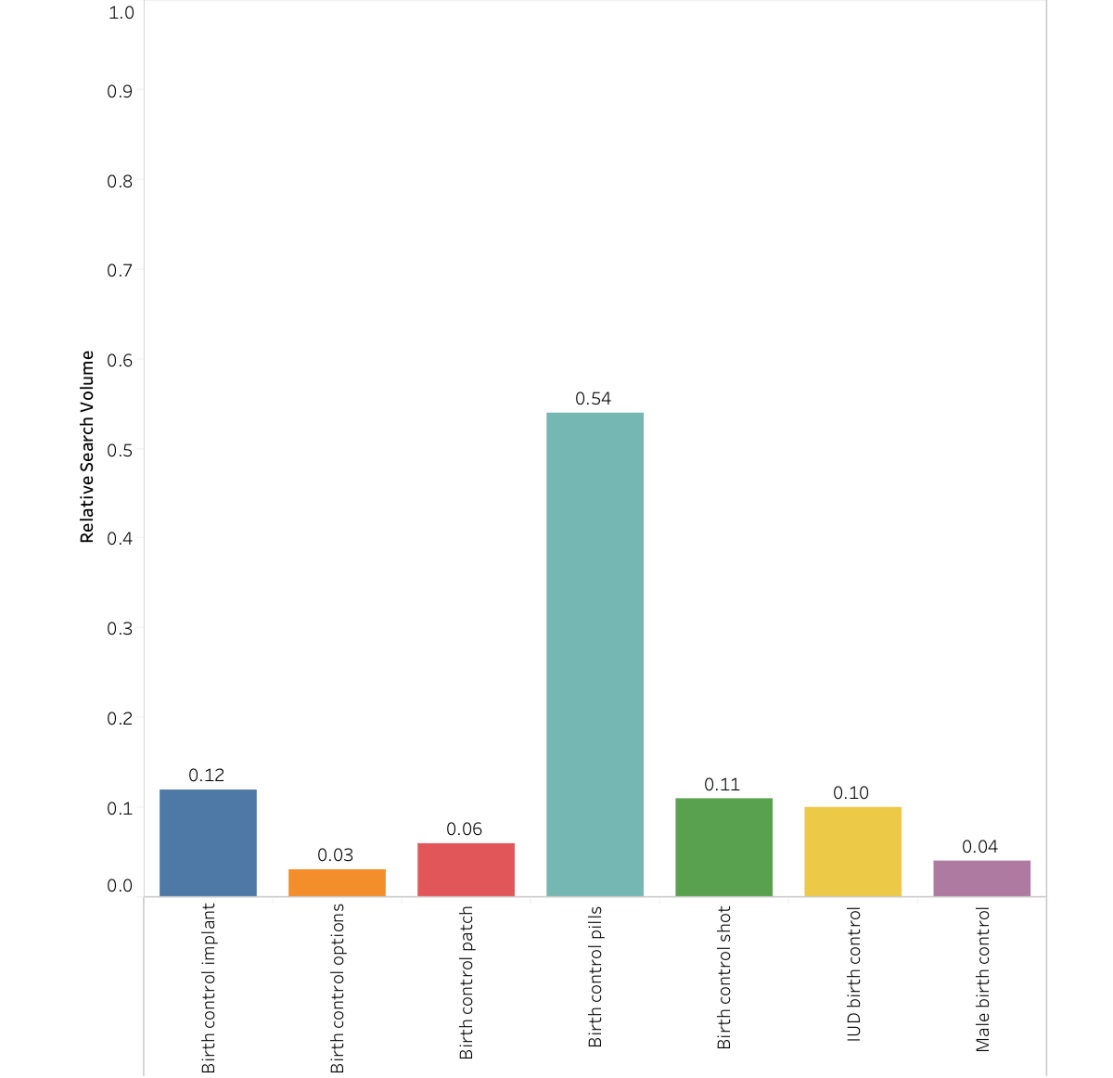
Relative search volume for *birth control* in the United States in 2017. IUD: intrauterine device.

In the second step, the *getTimelinesForHealth* function in the Google Health Trends API gives the RSV for the top search queries generated in the previous step. All values generated are relative: the API gives the relative frequency of a specific term as compared to the other terms in the *master list* for a specific region during a specified period of time. More step-by-step details are shown in Table 2. Figure 3 illustrates the normalized RSV as described in the intermediary Step 2.3.

**Table 2:** Gathering information on search volume.

Step	Description
2.1	For each region, for every term in the master list, send a request to the *getTimelinesForHealth* function from the Google Health Trends application programming interface (API) to obtain relative search volume. Figure MA2-4 in Multimedia Appendix 2 shows an example of the API call in Python.
2.2	The process in Step 2.1 should be repeated 30 times to minimize error. We take the average of the 30 samples of relative search volumes, which represents the estimated search volume for a given term with the date and location restrictions provided. Figure MA2-5 in Multimedia Appendix 2 shows a sample response of relative search volumes given by the *getTimelinesForHealth* function in the United States.
2.3	To compare across regions, normalize the values for each region by dividing each term’s value with the aggregate search volume for the region. Before normalization, the value returned is skewed and does not take into account parameters such as geographical size differences. The normalized value will range from 0 to 1. The total sum of all values of the set of queries is 1 after normalization. The value from 0 to 1 allows for understanding of the relative search frequency within search queries. These data can then be used to define search frequencies for each term (see Figure 3). The normalization function used in this study is found in Figure MA2-6 in Multimedia Appendix 2.

### Step 3: Determining the Most Popular Sites

In the third step, we send a request to the Google Custom Search API for each query in the master list to obtain a list of ranked top websites as they appear on the Google search engine results (see Table 3). A study from Chitika [39] demonstrated that the first 10 sites in the search results receive about 95% of the traffic or more, prompting us to only consider the first page of sites returned in the search results. According to the Chitika study, the probabilities of someone clicking on the first, second, and third sites are 0.35, 0.20, and 0.15, respectively; the probabilities of someone clicking on the fourth and fifth sites are 0.08 and 0.07, respectively. The probabilities keep decreasing, such that the probability of someone clicking on any site following the ninth site is 0.01. The Chitika frequencies for site rankings, the Custom Search API rankings, and the RSV for the query can be used to calculate the estimated proportion of site visits at the time of API access. The call to the Custom Search API is outlined in Figure MA2-7 in Multimedia Appendix 2. An example of this step, involving the term *birth control pills* and the top five sites visited [40-44], is illustrated in Table 4 in the Results section.

**Table 3:** Determining the most popular sites.

Step	Description
3.1	Use the master list generated in *Step 1: Developing a List of Search Queries* and send a request to the Google Custom Search application programming interface (API) for every term in the master list. This API returns a list of ranked top websites as they appear on the Google search engine results.
3.2	Use the frequencies for site rankings, the Custom Search API rankings, and the relative search volume for the query to calculate the estimated proportion of site visits. For example, as shown in Table 4, the top site for the term *birth control pills* in the United States is the *birth control pill* webpage on the Planned Parenthood website [40]. The relative search volume for *birth control pills* in the United States in 2017 is 0.54, and the probability of someone clicking on the first site returned on the Google search engine is 0.35. Thus, the estimated proportion of site visits to Planned Parenthood is 0.19.

**Table 4:** Top five sites visited for birth control pill searches in the United States in August 2018.

Site ranking	Website	Webpage
1	Planned Parenthood [40]	Birth control pill
2	WebMD [41]	Birth control pills
3	Wikipedia [42]	Combined oral contraceptive pill
4	BirthControl.com [43]	Birth control pills
5	Healthline [44]	Birth control pills: Are they right for you?

### Step 4: Calculating Estimated Total Search Volume

Google does not provide total search volumes. We overcome this limitation by using actual volume on searches to a concrete website as the baseline for calculating estimated total search volume corresponding to the RSV for the top search queries obtained from the Health Trends API. We worked with Planned Parenthood Federation of America (PPFA) to obtain the number of searches that led to their website, as this is the most popular website for reproductive health information that people access in the United States. PPFA works with Vector Media to collect analytics on the number of visitors to their site. A search is defined as a user typing in a query in a search engine and then being directed to the search engine’s results [45]. All of the data on searches that we obtained are, thus, the result of a user entering in a query regarding a particular initial search term in the Google search engine, which then leads them to the Planned Parenthood website. Slightly different processes must be used when the search query that directly relates to the site that the search data comes from is not present in the list of top queries. Estimated total search volume should be presented as a range that includes an upper bound determined by the lowest association of the top queries obtained. This assumes that there may be queries with lower associations that are not returned by the API. We show an example in the Results section.

## Results

### Step 1: Developing a List of Search Queries

We follow Step 1 of our procedure to gain information on the top queries for *birth control* in the United States in 2017. As shown in Figure 2, the most popular query was for birth control pills, followed in order of popularity by the shot, often searched for by its medical term Depo Provera and its effects; the implant; male birth control; and the intrauterine device (IUD). Queries for the IUD were predominantly for the copper IUD.

### Step 2: Gathering Information on Search Volume

We then use our findings in Step 1 to complete Step 2 of the protocol: determining the RSV of the top queries. Figure 3 shows, for instance, that in the United States in 2017, the pill (RSV=0.54) was searched for 4.5 times more than the implant (RSV=0.12) and 5.4 times more than male birth control (RSV=0.04).

### Step 3: Determining the Most Popular Sites

We follow Step 3 of the protocol to obtain information on top sites. We chose one top query, *birth control pills*, as an example to demonstrate; however, to gain a full picture of top sites viewed, it is important to carry out this step for all top queries (see Table 4).

### Step 4: Calculating the Estimated Total Search Volume

**Figure 4:**
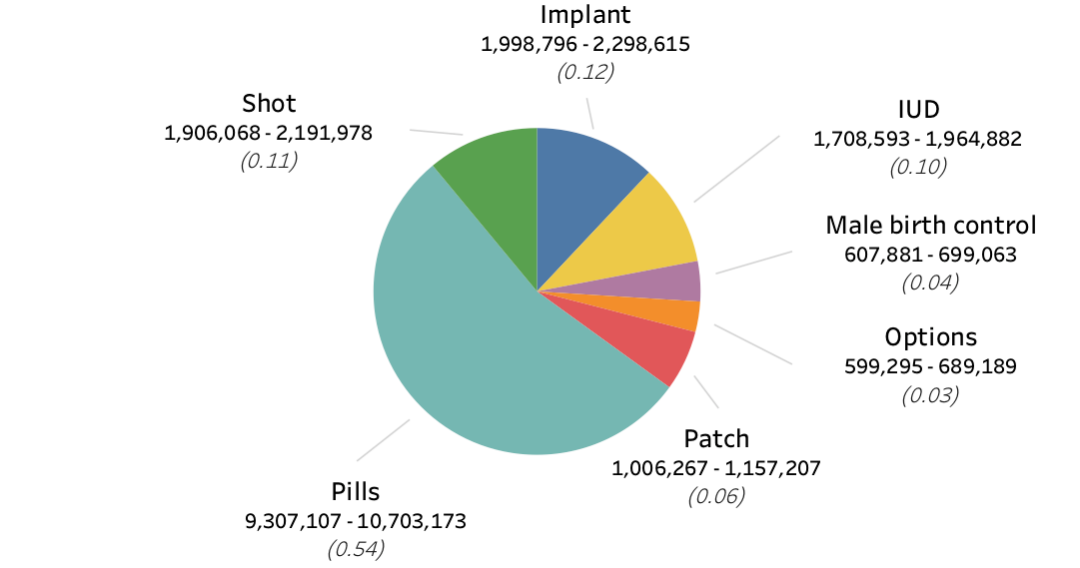
Estimated total search volume (range) and relative search volume (within parentheses) for *birth control* in the United States in 2017. IUD: intrauterine device.

We estimate that the total number of searches for birth control in 2017 fell within the following ranges for the United States: 17,171,784-19,747,552 searches. These values were calculated using the formula outlined in Table 5. *Planned Parenthood* is not a top search query for the term *birth control*, but as we found out, it is a top search query for *abortion*. By obtaining the RSV of *birth control* as compared to *abortion*, we were able to obtain the estimated total search volume for *birth control* and then applied the RSV weights to obtain estimated total search volume for the top queries. Because the top queries do not account for all queries searched for—evidenced by the association values presented in Figure 3—we calculated an upper bound of 15% that we include in our estimates. Figure 4 shows the estimated total search volume for each of the top search queries for *birth control* in the United States in 2017 based on the RSV weights for the top birth control methods.

**Table 5:** Calculations for estimated total search volume for birth control in the United States.

Search item measure	Value
Total number of searches for *abortion* from Planned Parenthood	12,393,960
RSV^a^ for *abortion*	0.4192
RSV for *birth control*	0.5808
Estimated total number of searches overall	29,565,744^b^
Estimated total number of searches for *birth control*	17,171,784^c^

^a^RSV: relative search volume.

^b^0.4192 (*RSV for abortion*)x = 12,393,960 (total number of searches for *abortion* from Planned Parenthood); x = 29,565,744.

^c^0.5808 (RSV for *birth control*) × 29,565,744 (estimated total number of searches overall) = 17,171,784.

## Discussion

### Principal Findings

Google Trends has become a popular tool for analyzing search traffic on health. It has been used by researchers to measure general web-based interest, examine policy-related issues, get insights into health behavior, and to monitor and predict health-related events [27,29]. However, it has been used inconsistently due to a lack of consensus on how to document Google search engine queries in academic research. This leaves room for methodological development. In this article, we show that Google Trends data, when retrieved from the Google Trends API, offers more versatile analytic capabilities than the data from the Google Trends website and offers the benefit of incorporating other APIs to extend insight into search-traffic behavior.

The proposed protocol—empirically tested with *birth control* as the key initial search term—is capable of addressing important questions about Google search traffic and search interest. By following four distinct steps using three Google APIs, we are able to identify top search queries and websites as well as estimate relative and total search volume of queries in real time or near-real time in specified locations and time periods. The use of multiple APIs also provides multiple methods to obtain key values, ensuring the data obtained are accurate and reliable.

Our methodology is robust insofar as it is well documented and avoids inserting any personal bias into the process of determining top search queries, since all top queries are given by the API. In addition, we are able to provide a novel solution to the current limitation of Google data, which, for privacy concerns, does not provide the absolute volume of searches. Prior studies proposed an approach to calculating total estimated search volume [25], but this approach is no longer replicable given the constant updates Google makes to its technologies.

The thorough documentation provided to apply the proposed protocol will allow researchers to replicate the methods used to further the understanding of population interest in health issues. The protocol can be applied to compare state-level searches to those at the national level and to explore changes in search traffic over time. It can easily be applied to other initial search terms; in our own exploration, we found that in the United States, people who searched for *family planning* instead of *birth control* were searching for traditional or *natural family planning* methods based on fertility awareness. Additionally, our protocol can be utilized at the zip code–based Nielsen Demographic Marketing Area (DMA) level, which is the smallest geolocation level that Google reports on and is available for each state. However, to protect user privacy, Google does not report data below a certain unknown threshold, so data may be unavailable for some DMAs.

Google data can provide essential context to administrators, health care professionals, and academics. Top queries show varying interest in health topics as well as products and services by location, thus allowing health care providers to tailor services and information available at clinics and local practices to the questions people are asking. RSV provides context on how search interest compares by location, thus allowing one to focus on what resources are most desired or sought out. Top websites are crucial information for researchers, as they give a direct picture of what searchers are finding when they seek information. This data can provide insight as to why misinformation may spread or what organizations are having the greatest influence in sharing their beliefs, products, and services with potential patients and/or consumers. Finally, estimated total search volume allows professionals to know the amount of the population that may be seeking access to resources or information on the resources in question. More broadly speaking, this data gives interested stakeholders understanding of the changing health care landscape and identifies key concerns of potential patients and clients. Trends in search data over time may reveal the impact of administrative revisions and/or decisions made at the state or national level.

### Limitations

The results that our protocol can achieve must be tempered by the limitations of the data and the data sources. Google Trends reports on the top related and rising queries, as well as the top related and rising topics, but does not provide a list of all queries searched for. Thus, although the list of top queries is a comprehensive list of the most popular queries that users search for, it does not include every single query searched for relating to a particular initial search term. Similarly, the RSV is only relative to the other queries in our final list and does not include other queries that were not a part of the list of top queries. Furthermore, we are not able to identify the number of unique users or their individual characteristics.

For most popular sites, we were unable to identify the key websites at the state level or request a specified time period. To overcome this limitation, one could import another source of data, such as a Google Consumer Survey (GCS) run at the state level. GCS is a tool that allows for online, customized market research and can be used to survey internet users about their preferred websites that they seek for specific queries [46]. The values obtained from these responses could additionally be used as anchor points for calculating total volume of searches.

Clearly, we require more studies to assess the value and validity of the proposed methodology. Temporal changes in the interface and capabilities of Google data pose challenges to the research community because researchers cannot build on nonspecific, nonreplicable, and discontinued methodologies. Hence, the proposed methodology will necessarily evolve as Google continues to make changes. In June 2019, Google made additional changes to the Google Trends API that had an effect on the *getTopQueries* function, resulting in a broader list of top queries than when our study data were retrieved. Future studies may integrate Google searches and other sources of online big data with machine learning models to track health topics [47].

### Conclusions

The combination of Google APIs suggested in the proposed methodological framework offers a novel approach to analysis of Google health queries, expanding the tools available to gain insight into health assessments.

## Appendix

Multimedia Appendix 1 Google Trends website and Google Trends application programming interface (API) comparison.

Multimedia Appendix 2 Python code appendix.

Multimedia Appendix 3 Details of packages used within code and documentation for application programming interfaces (APIs).

## Abbreviations

API: application programming interface
CSV: Comma-Separated Values
DMA: Demographic Marketing Area
GCS: Google Consumer Survey
IUD: intrauterine device
JSON: JavaScript Object Notation
PPFA: Planned Parenthood Federation of America
RSV: relative search volume
