# Supplementary material for: Using Application Programming Interfaces to Access Google Data for Health Research: Protocol for a Methodological Framework
Source: JMIR Res Protoc. 2020 Jul 6;9(7):e16543. doi: 10.2196/16543 (PMC7381000; doi:10.2196/16543)
Supplement: Multimedia Appendix 1 [file resprot_v9i7e16543_app1.docx]

## **Multimedia Appendix 1: Comparison of Google Trends Website with Google Trends API**

The Google Trends API presents the same set of information as provided by the Google Trends website; the Google Trends website gives this data in the form of visuals and CSV files, while the API gives raw data in the form of JavaScript Object Notation (JSON). The Google Trends website home page – the “Explore” page -- allows researchers to enter a search term of interest, and then additional keywords, specified location/region (e.g. country, state, designated marketing area), and category to view search data in a given time period (e.g. daily, monthly, yearly, etc.). Google classifies search queries into 25 categories of which health is one.

Figure 1 of the appendix illustrates the website’s detailed landing page after entering the “am i pregnant” for the United States in 2017. Note that Google Trends is not case sensitive. The website displays a graph with the relative search interest for “am i pregnant” for a random sample of monthly data points. The weekly relative search index (a measure of search interest) is shown when hovering over the graph (Figure 2). Google Trends expresses the relative popularity of a search term by calculating the total number of searches for a specific search term relative to the total number of searches in each location and at each time period. The resulting proportions are scaled on a range of 0 (least popular) to 100 (most popular). A 100 search index represents the month that saw the highest relative search interest (i.e. highest volume of search) for “am i pregnant”, while an index of 82 for example (Figure 2), indicates a search activity for “am i pregnant” that is 82% of that seen when the search activity was most intense. Additionally, the website provides the relative search index by region as displayed in a heatmap (Figure 1) and in a listing (Figure 1). In the case of the United States, each state (or sub-region) for which there is enough data to meet Google’s minimum threshold is included. If data fall below the minimum threshold to maintain confidentiality, Google reports the search index as 0. All data can be downloaded as CSV files. The website also gives the top and rising queries and topics (Figure 3). The values for rising queries and topics represent the percentage of rising searches that had the most growth in volume during the selected time-period since the previous time range (Figure 4). The values for top queries and topics represent the most frequently searched queries or topics with the initial keyword entered in the same search session within the chosen category and region. For all the data displayed, these values are a sample of the search data during this time-period and should be treated accordingly.

**Figure 1**: Google Trends Website Detail Page for “am i pregnant” in the United States in 2017


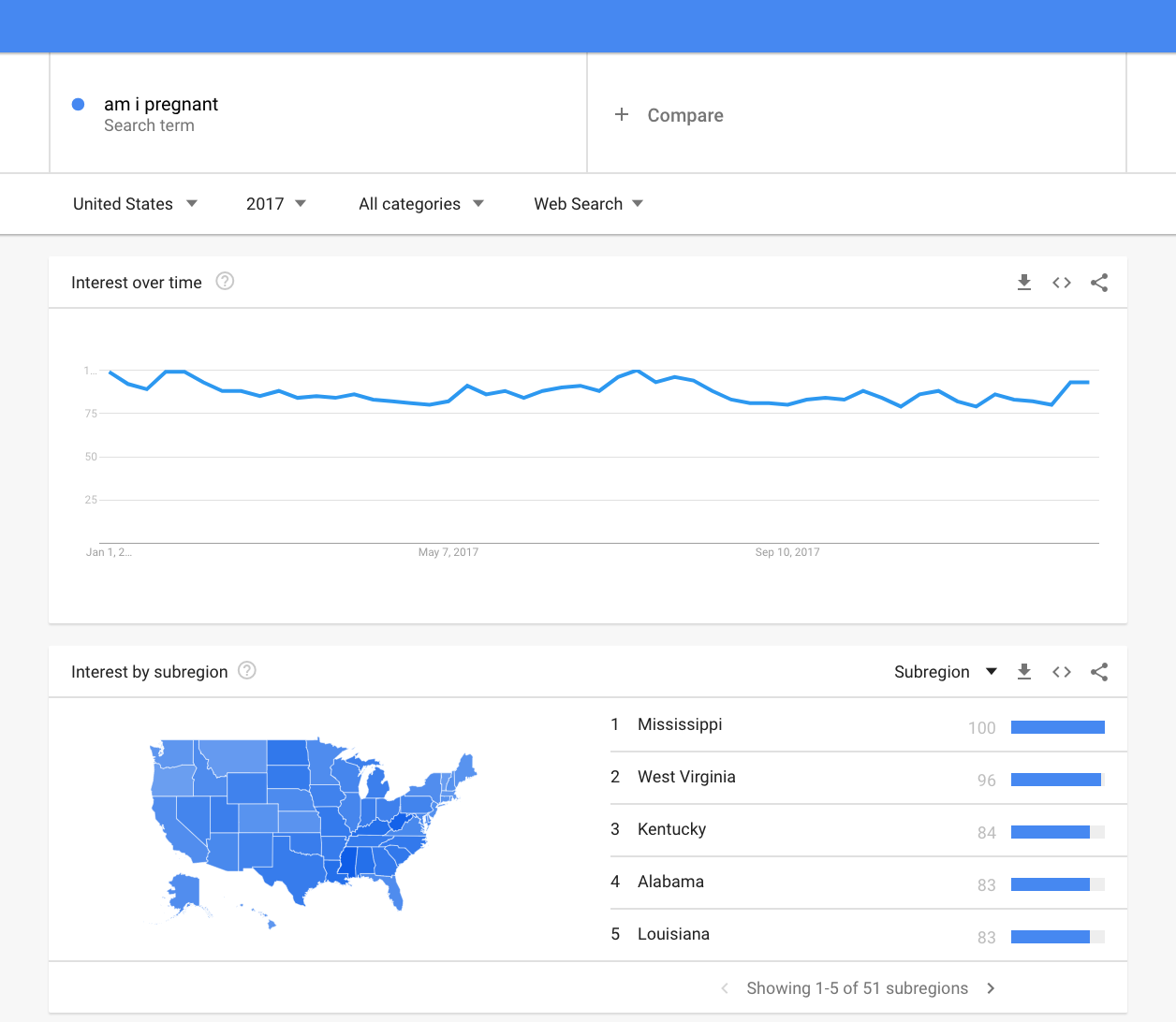


**Figure 2**: Google Trends Search Index During May 7-13 2017 for “am i pregnant” in the United States


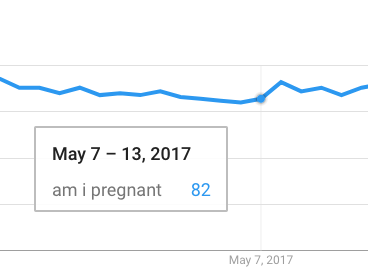


**Figure 3**: Top Topics and Top Queries for “am i pregnant” in the United States in 2017


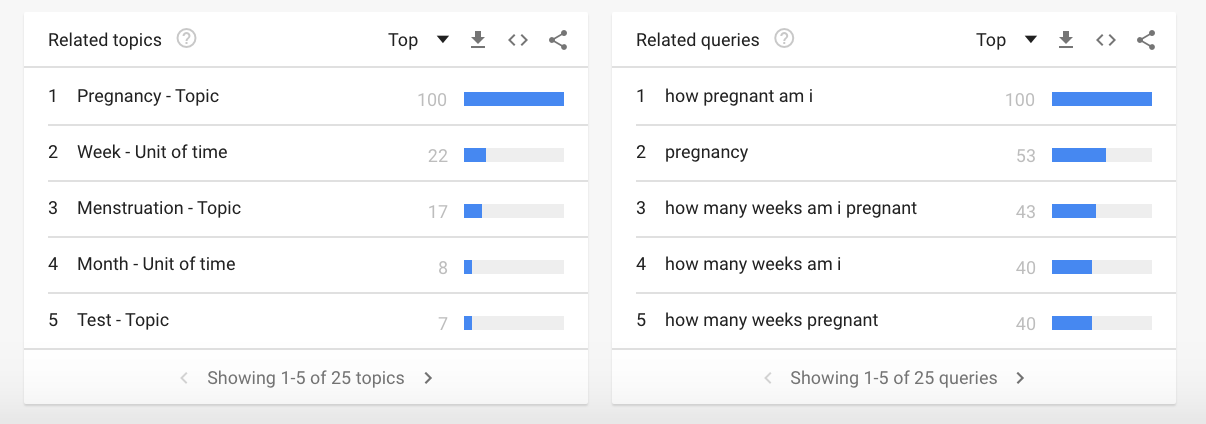


**Figure 4**: Rising Topics and Rising Queries for “am i pregnant” in the United States in 2017


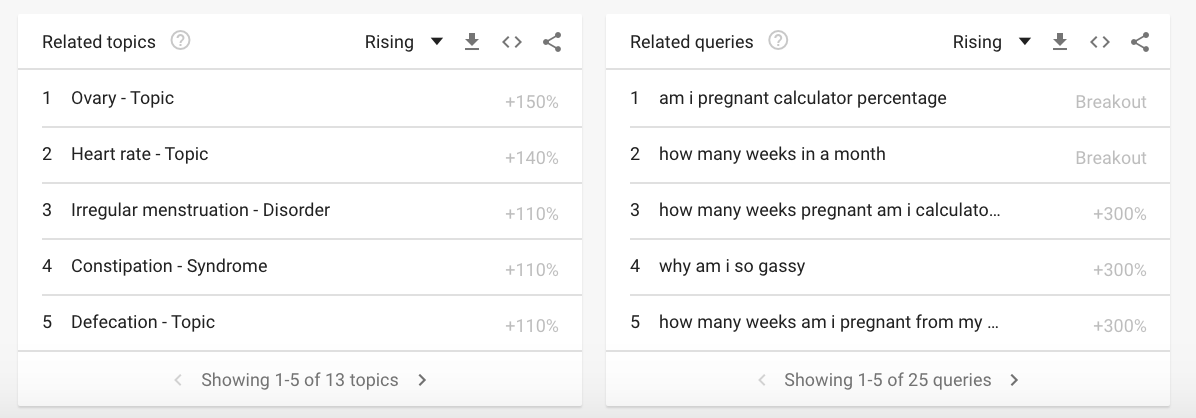


With the Google Trends API, researchers can obtain the same type of data for a search query. Researchers may choose a search term of interest and may set restrictions to obtain sample data on that term in a specified location during a specified time-period. For ease of comparison we will also examine the search term “am i pregnant” for the United States in 2017. The API function “getGraph” obtains a sample of data points on a month-by-month basis for the relative search interest during the defined period of time and location (Figure 5) with which we can create a graph (Figure 6) which is similar to that generated by Google Trends website in Figure 1. The API also allows us to obtain relative search index data for specific sub-regions (same regions as offered in the Google Trends website); this can be done through the “regions” function (Figure 7). We can see that so far, the data obtained from the API are similar to that presented by the Google Trends website. The data available continue to be comparable, as the Google Trends API also allows users to obtain the top and rising queries (Figure 8) and topics (Figure 9) for a search term. Data differences between the two tools may result from sample differences since Google only provides a sample of data each time the user accesses them.

**Figure 5**: JavaScript Object Notation (JSON) Output from “getGraph” Google Trends API call in Python for “am i pregnant” in the United States in 2017

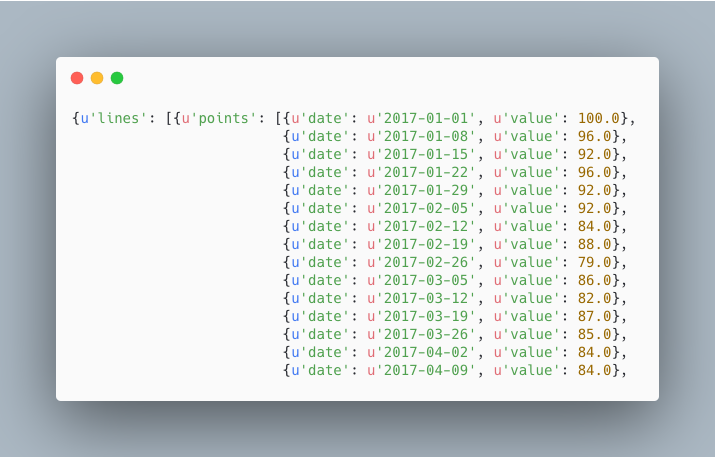


**Figure 6**: Interest Over Time from Google Trends API for “am i pregnant” in the United States in 2017

**Figure 7**: JavaScript Object Notation (JSON) “regions” Sample Data for “am i pregnant” in 2017 in the US


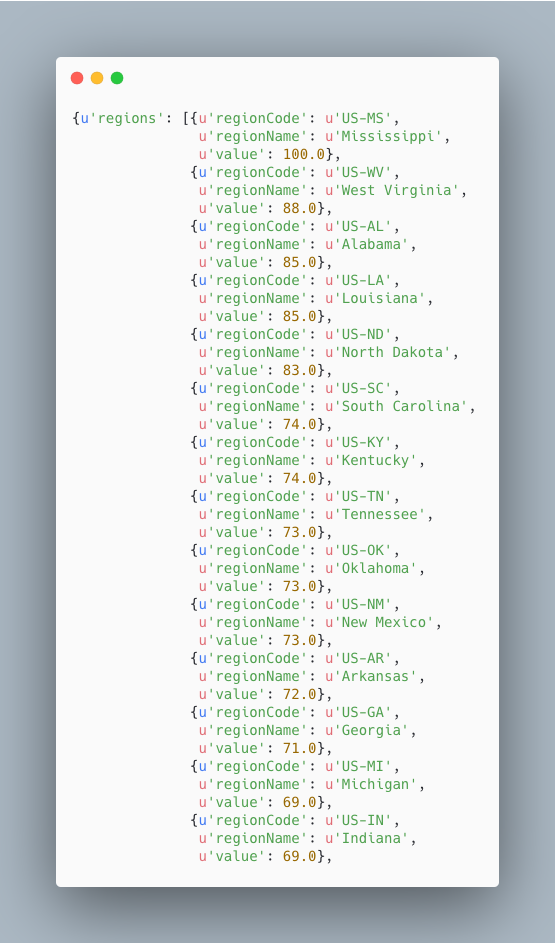


**Figure 8**: JavaScript Object Notation (JSON) Top Queries Sample Data for “am i pregnant” in 2017 in the US


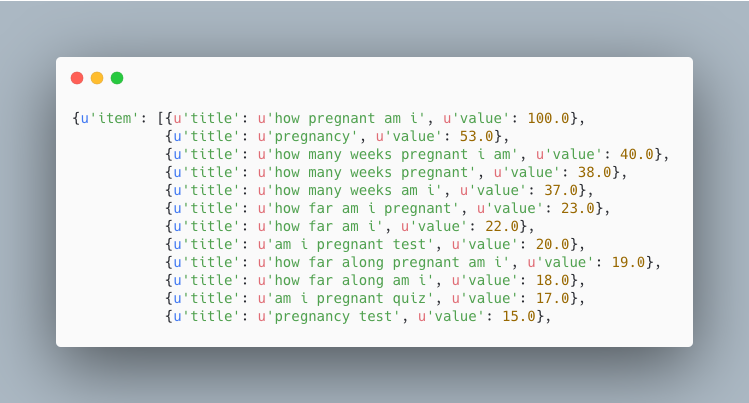


**Figure 9**: JavaScript Object Notation (JSON) Rising Topics Sample Data for “am i pregnant” in 2017 in the US


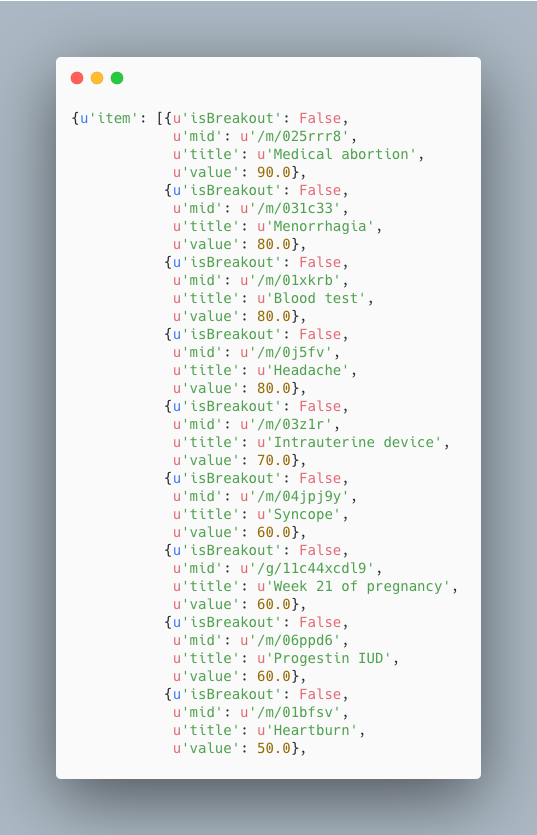


It is easier to make relative comparisons among terms or across time and/or location using the Google Trends API. With the website, the user must manually enter the locations of interest and is limited to five locations on one web page whereas with the API, there is no such limit. A user can quickly obtain data for a set of regions by running all functions for all the specified regions. Another advantage of the API is the ability to filter data. Top queries and topics can be influenced over time by media coverage which lead people to seek online information on these issues. For instance, when people search for “pregnant” they may be interested in health issues such as “can you get pregnant with an IUD” or “can you take allergy medicine when pregnant”. Alternatively, they may be curious whether a celebrity is pregnant. Using a filter helps ensure that “noisy” queries are removed from the final datasets.
