# Supplementary material for: Using Application Programming Interfaces to Access Google Data for Health Research: Protocol for a Methodological Framework
Source: JMIR Res Protoc. 2020 Jul 6;9(7):e16543. doi: 10.2196/16543 (PMC7381000; doi:10.2196/16543)

**Figure MA2-1**: JavaScript Object Notation (JSON) Top Queries Sample Data for “birth control” in 2017 in the US.


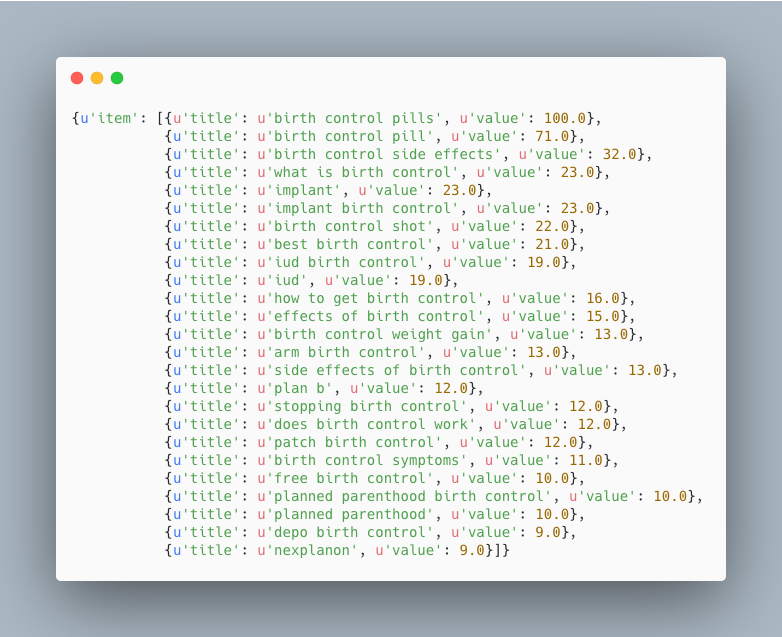


**Figure MA2-2**: Python Snippet of API Request to “getTopQueries” function in Step 1: Developing a List of Search Terms.


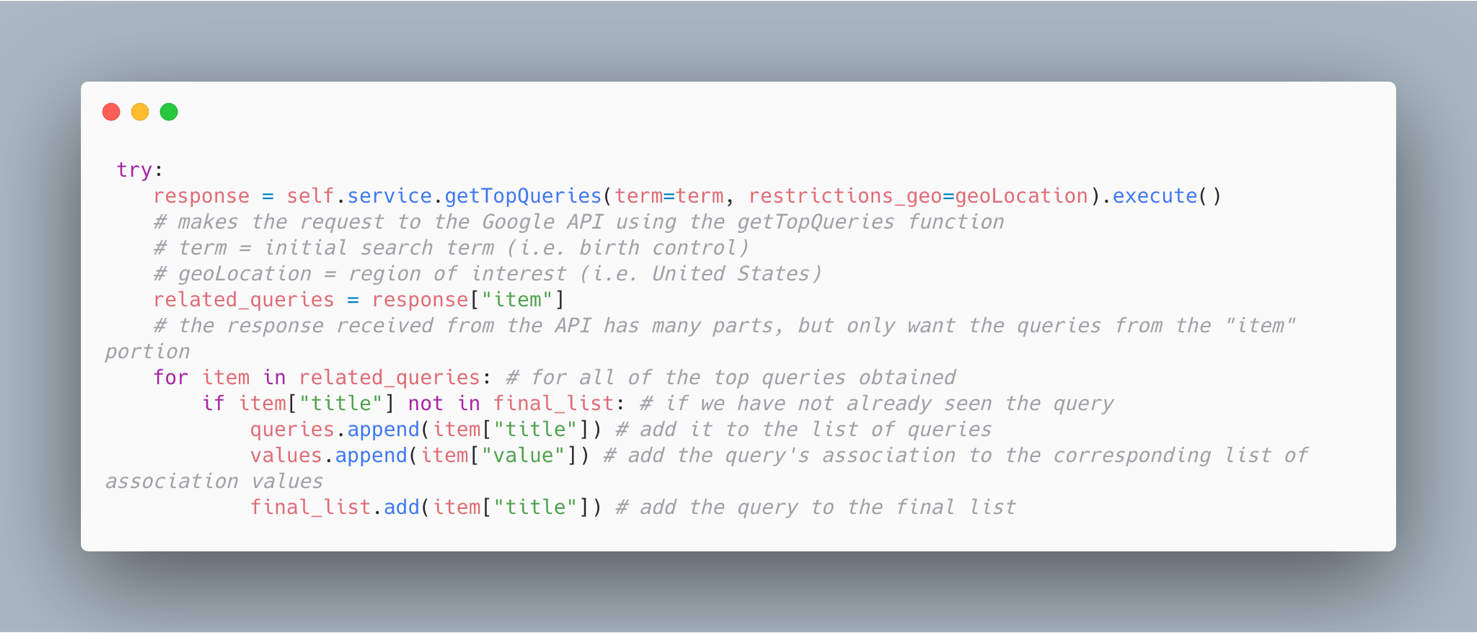


**Figure MA2-3**: Python Snippet of Graphviz Bubble Graph Generation in Step 1: Developing a List of Search Terms.


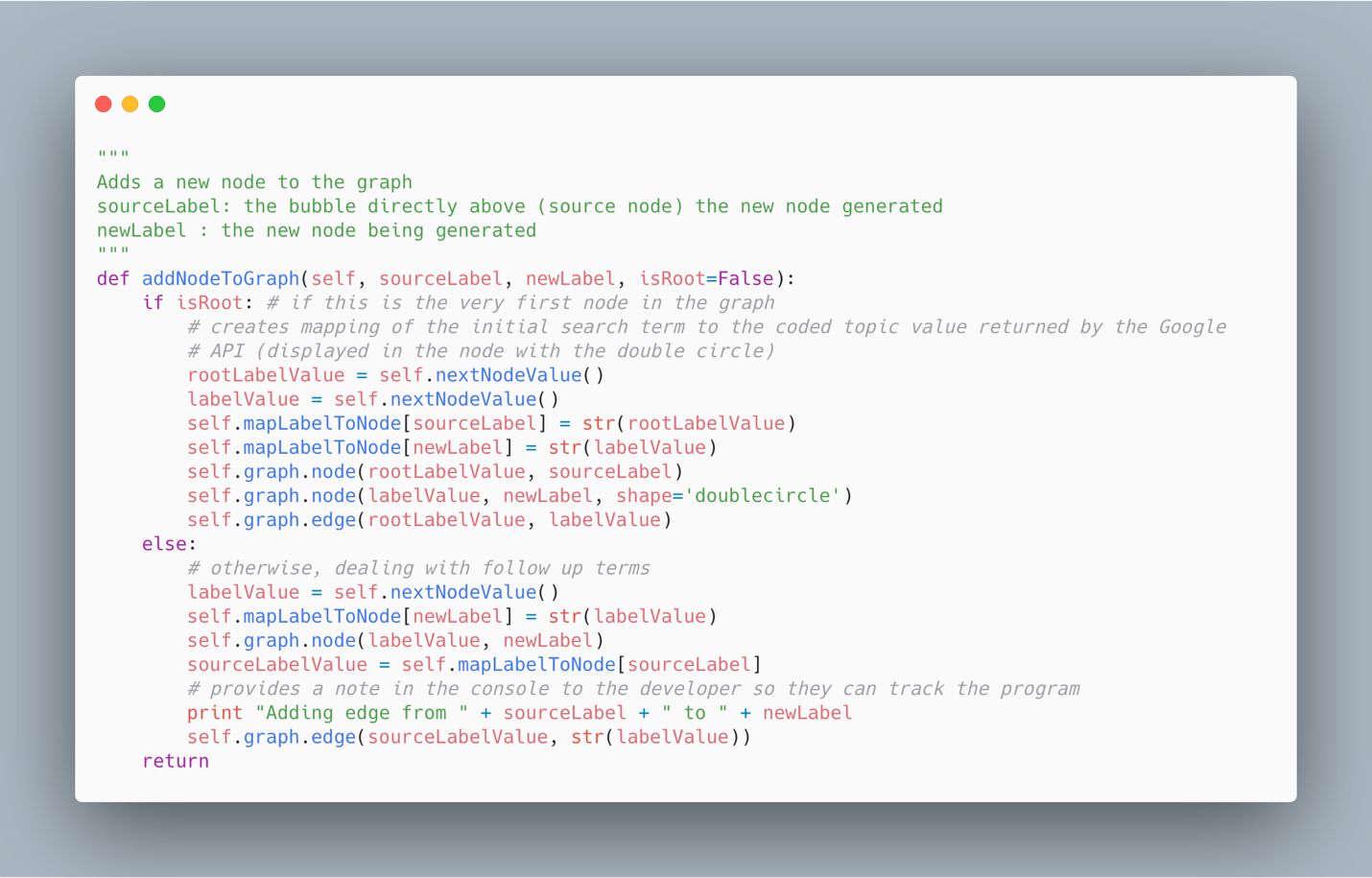


**Figure MA2-4**: Python Snippet of “getTimelinesForHealth” API call in Step 2: Gathering Information on Search Volume: Custom HealthTrends API.


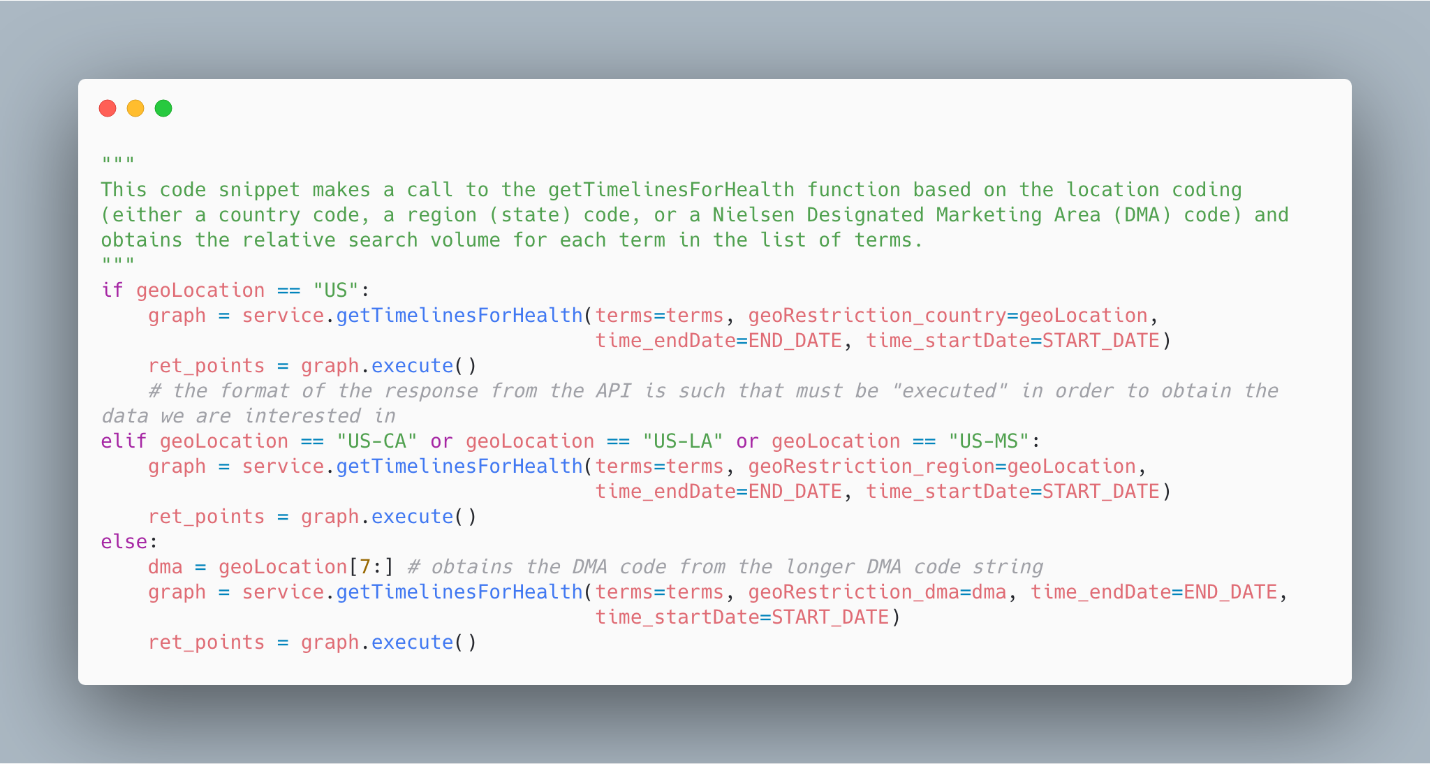


**Figure MA2-5**: Sample Response from run of “getTimelinesForHealth” function for birth control top queries in United States from 01-01-2018 to 12-31-2018.


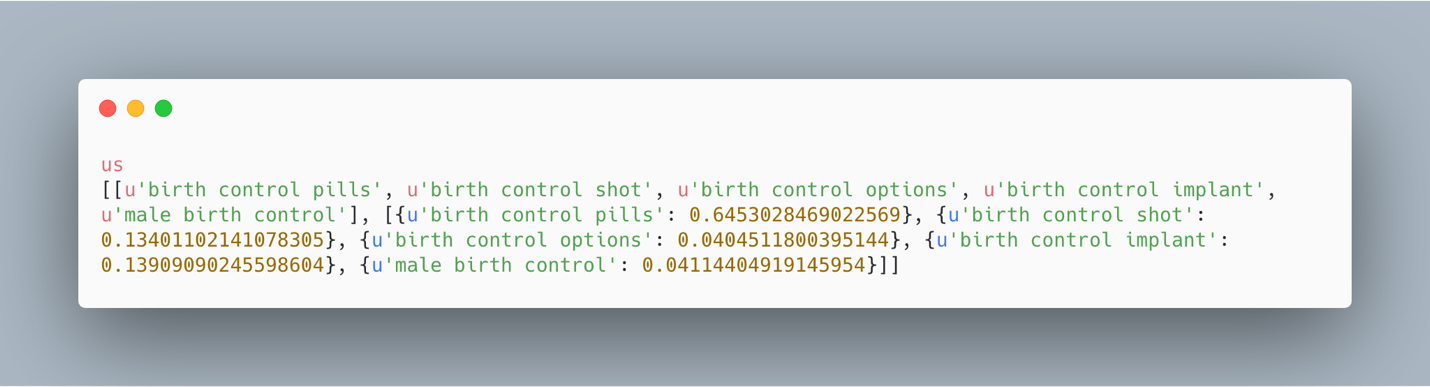


*Note: the ‘u’ in the response means that the strings were converted to Unicode*

**Figure MA2-6**: Normalization function in Python in Step 2: Gathering Information on Search Volume: Custom Health trends API.


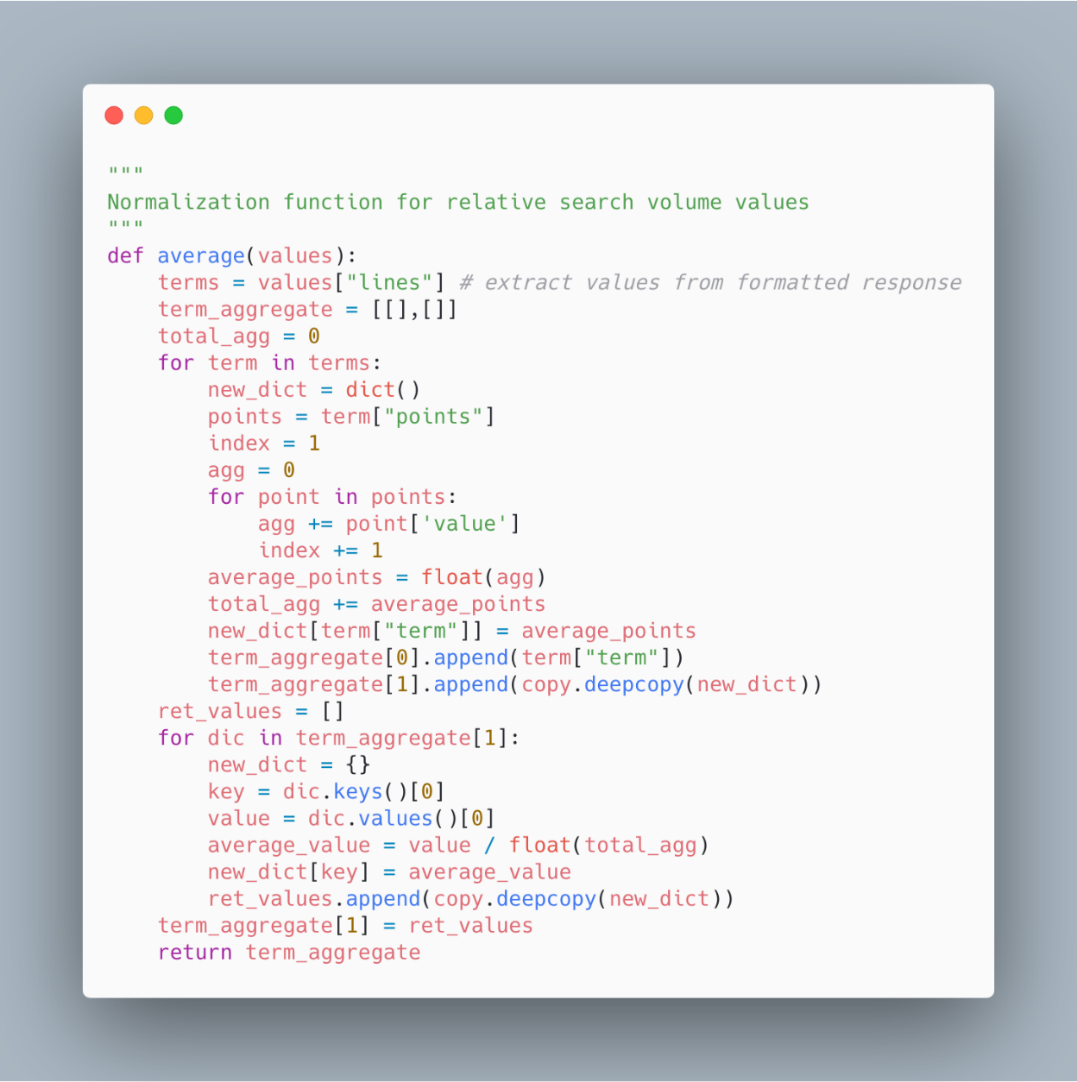


**Figure MA2-7**: Python Code Snippet for Custom Search API Call in Step 3: Determining Most Popular Sites.


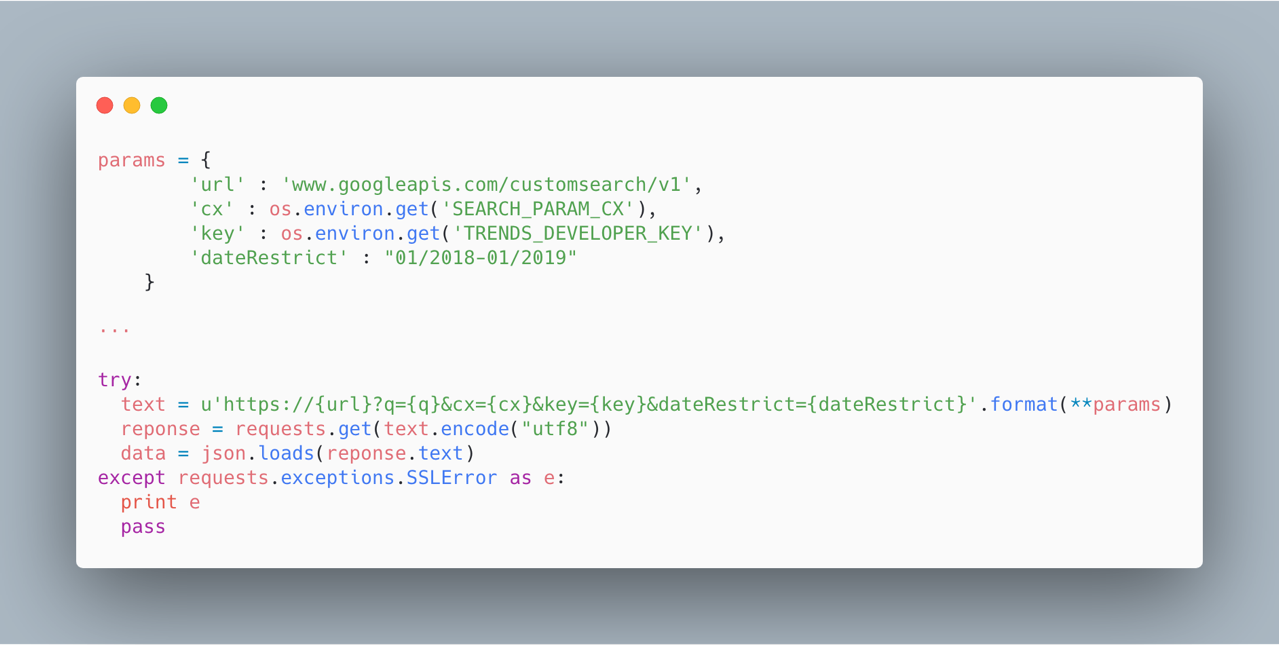

Supplement: Multimedia Appendix 2 [file resprot_v9i7e16543_app2.docx]
