# Supplementary material for: Using Application Programming Interfaces to Access Google Data for Health Research: Protocol for a Methodological Framework
Source: JMIR Res Protoc. 2020 Jul 6;9(7):e16543. doi: 10.2196/16543 (PMC7381000; doi:10.2196/16543)
Supplement: Multimedia Appendix 3 [file resprot_v9i7e16543_app3.docx]

**External Packages:**

Graphviz 0.13:

*GitHub Source Code:* <https://github.com/xflr6/graphviz>

*Documentation:* <https://graphviz.readthedocs.io/en/stable/>

Google Custom Search API :

*Documentation :* <https://developers.google.com/custom-search/docs/overview>

Google Trends API :

*Public Site :* <https://trends.google.com/trends/?geo=US>

*Documentation:* <https://docs.google.com/document/d/1Ybu3gHUHtcSXXzgDJ-m7PPto9tw0QG8A5oOBsFP2jao/edit>

Google API Python Library :

*GitHub Source Code :* <https://github.com/googleapis/google-api-python-client>
